# 638 The Teen-Aged Life Impact Burn Recovery Evaluation: New and Existing Perspectives on “Experience of Self”

**DOI:** 10.1093/jbcr/iraf019.267

**Published:** 2025-04-01

**Authors:** Yasameen Farahvash, Tina Palmieri, Madeleine McGwin, Sophia McLaughlin, Khushbu Patel, Alexandra Gladstone, Ludwik Branski, Mary Slavin, Michael Murphy, Frederick Stoddard, Jeffrey Schneider, Lewis Kazis, Colleen Ryan

**Affiliations:** Shriners Children’s Northern California; University of California, Davis and Shriners Hospitals for Children; Shriners Children’s – Boston; Shriners Children’s – Boston; Boston Medical Center; Northeastern University; University of Texas Medical Branch; Spaulding Rehabilitation Hospital; Shriners Children’s – Boston; Harvard Medical School; Spaulding Rehabilitation Hospital, Harvard Medical School; Boston University; Shriners Children’s - Boston and Massachusetts General Hospital

## Abstract

**Introduction:**

Between the ages of 12 and 19, teenagers explore unique identities and solidify a sense of self. These formative experiences become even more challenging for teen burn survivors, as they recover from injury and navigate altered perceptions of themselves. To better understand the impacts of burn-related injuries on teenagers, the Teen-Aged Life Impact Burn Recovery Evaluation (TA-LIBRE12-19) survey was developed using a conceptual framework from the World Health Organization’s International Classification for Functioning, Disability, and Health for Children and Youth, supplemented with existing instruments and focus group feedback. The purpose of this analysis is to compare the “Experience of Self” items, or questions, derived from our focus groups, with the “Experience of Self” items from existing, established instruments.

**Methods:**

Our analysis of existing instruments included items from general quality of life instruments and burn-specific outcome assessment instruments for teens. We conducted focus groups with eligible teens, and/or their representative parents, where participants identified the health outcomes that they felt were most relevant to assessing recovery. We encouraged participants to speak about the perceived impact of the burn injury on various physical, psychological, and social health factors. Following focus group transcription, we identified emergent themes using NVivo software. Subsequently, we analyzed the data via descriptive statistics.

**Results:**

From our preliminary analyses, we identified over 1,700 items from existing instruments corresponding to the “Experience of Self.” Our four focus groups gave us insight into the lived experiences of eight total teen burn survivors. Median age among the teens was 14.2 years old (IQR 12.1-16.3), with 62% identifying as female. 50% of burn injuries occurred due to fire/flame, with a median burn size of 20% TBSA (IQR 0.3 – 60) and a median time of 6.3 years (IQR 0.6 – 13.7) elapsed since injury. Regarding the survey, 39.7% (n=29) of the items related to the “Experience of Self” were developed from our focus groups, while 60.3% (n=44) of the items were from existing surveys. Over 40% of the new items from the focus groups were related to negative affect and scar appearance.

**Conclusions:**

Over a third of survey items were developed from the focus groups. Teenagers’ responses about their experiences provide unique perspectives on their burn injury recovery that add depth to existing instruments.

**Applicability of Research to Practice:**

Current instruments do not capture the entirety of what is important to teen burn survivors. Modification of existing tools is necessary for accurate representation of a teenager’s ever-evolving development. We will incorporate this work into the preliminary item pool for field-testing of a burn-specific and age-specific outcome tool.

**Funding for the Study:**

This work was funded by Foundation Funding (Grant #79136) and partial support from the National Institute on Disability, Independent Living, and Rehabilitation Research (Grant #90DPBU0008).

